# Genome-wide transcriptome analysis of gametophyte development in *Physcomitrella patens*

**DOI:** 10.1186/1471-2229-11-177

**Published:** 2011-12-15

**Authors:** Lihong Xiao, Hui Wang, Ping Wan, Tingyun Kuang, Yikun He

**Affiliations:** 1 College of Life Science, Capital Normal University, Beijing, 100048, P. R. China; 2 Institute of Botany, Chinese Academy of Sciences, Beijing, 100093, P. R. China

## Abstract

**Background:**

Regulation of gene expression plays a pivotal role in controlling the development of multicellular plants. To explore the molecular mechanism of plant developmental-stage transition and cell-fate determination, a genome-wide analysis was undertaken of sequential developmental time-points and individual tissue types in the model moss *Physcomitrella patens *because of the short life cycle and relative structural simplicity of this plant.

**Results:**

Gene expression was analyzed by digital gene expression tag profiling of samples taken from *P. patens *protonema at 3, 14 and 24 days, and from leafy shoot tissues at 30 days, after protoplast isolation, and from 14-day-old caulonemal and chloronemal tissues. In total, 4333 genes were identified as differentially displayed. Among these genes, 4129 were developmental-stage specific and 423 were preferentially expressed in either chloronemal or caulonemal tissues. Most of the differentially displayed genes were assigned to functions in organic substance and energy metabolism or macromolecule biosynthetic and catabolic processes based on gene ontology descriptions. In addition, some regulatory genes identified as candidates might be involved in controlling the developmental-stage transition and cell differentiation, namely MYB-like, HB-8, AL3, zinc finger family proteins, bHLH superfamily, GATA superfamily, GATA and bZIP transcription factors, protein kinases, genes related to protein/amino acid methylation, and auxin, ethylene, and cytokinin signaling pathways.

**Conclusions:**

These genes that show highly dynamic changes in expression during development in *P. patens *are potential targets for further functional characterization and evolutionary developmental biology studies.

## Background

Most plants originate from a single-celled zygote [1]. To achieve maturity, a plant must pass through different developmental stages that comprise a series of cell division, cell expansion and cell differentiation processes. Despite the practical difficulties, understanding the regulation of plant development is reasonably well advanced for some processes, including lateral root formation, seed development, and fruit development, and genes that regulate these processes have been identified [2-6]. However, we are still far from fully understanding the molecular mechanisms that govern development.

The increasing power of genomic tools enables the changes in expression of thousands of genes to be profiled in parallel during sequential developmental stages. Several high-throughput techniques can be used to study expression of mRNAs, proteins, and metabolites [7]. Although the final activity of a particular gene is determined by the encoded protein, measurements of mRNA levels have proven to be valuable for identification of the molecular changes that occur in cells. Transcriptomic analysis could also provide clues for construction of a digital *in situ *hybridization map of the cellular systems or cell-to-cell networks [8].

We chose to identify regulatory developmental genes using the model bryophyte, *Physcomitrella patens*. Although they diverged hundreds of millions of years ago, bryophytes have similar fundamental genetic and physiological features to those of seed plants [9]. Compared to vascular plants, bryophytes are argued to be an ideal system to study developmental mechanisms because of their accessible haploid stage and relatively simple structure [10]. Among bryophytes, the moss *Physcomitrella patens *has emerged recently as the bryophyte model of choice for several reasons, including its short life cycle (about 3 months) [11] and efficient homologous recombination [12]. Furthermore, *P. patens *is placed phylogenetically between algae and seed plants [13]. Therefore, studies on this species might be of both developmental and evolutionary significance.

The life cycle of *P. patens *consists of two generations: the haploid gametophyte generation followed by the diploid sporophyte [13]. Gametophyte development is further divided into two stages: a juvenile filamentous stage, termed the protonema, and leafy adult stage, termed the gametophore because each leafy stem is capable of generating the sex organs [14,15]. The vegetative development of *P. patens *involves only a few cell types. In *P. patens*, when the haploid spore germinates, or when a protoplast derived from protonema tissue regenerates, a single-celled filament forms with the apical cell dividing and elongating by tip growth. Soon after the filament forms, subapical cells divide obliquely and give rise to branches, thereby forming a highly branched filamentous network (protonema). Initially, the protonema has perpendicular cross walls and is filled with large, spherical chloroplasts; filaments comprising this type of cell are called chloronema. Subsequently, a second filament type develops, often in response to light and auxin, called a caulonema [13]. Compared with chloronemal cells, caulonemal cells are longer, divide more often, and contain fewer chloroplasts, which are smaller and flattened. Finally, the kind of branches produced changes from protonemal to those that give rise to leafy gametophores [16]. While protonemata grow in a two-dimensional network, the leafy gametophores grow upwards toward the light, thus forming a three-dimensional gametophyte.

Recently, the availability of a high-quality genomic sequence for *P. patens *[9] has facilitated '-omic' studies on this organism. In this study, we used the digital gene expression tag profiling (DGEP) strategy driven by high-throughput sequencing technology to profile the transcripts at different stages of *P. patens *gametophyte development and obtained spatiotemporal-specific gene regulation models for growth and differentiation. Furthermore, we also compared mRNA profiles from chloronemal and caulonemal tissues, isolated with laser-capture microdissection. These results provide a comprehensive catalogue of gene expression changes from which potentially regulatory changes can be mined.

## Results

### Gametophytic morphogenesis during the culture of *P. patens *protoplasts

**Figure 1:**
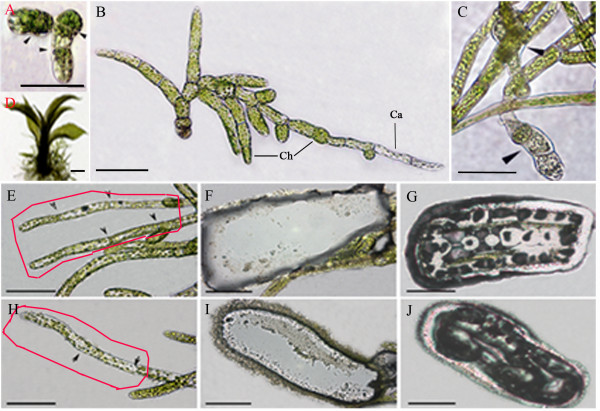
**Gametophyte development and cell isolation in *Physcomitrella patens***. (A) Plantlet on day 3 after protoplast formation (arrows indicate cell walls). (B) Plantlet on day 14 after protoplast formation. Ca = caulonema; Ch = chloronema. (C) Region of a plantlet on day 24 after protoplast formation (arrowheads show buds). (D) Plantlet on day 30 after protoplast formation. Plantlet contains leafy gametophores, protonema, and rhizoids (not visible in the image). (E-J) Images illustrating the process of laser-capture microdissection. Chloronema (E) and caulonema (H) before dissection. Arrowheads indicate cell walls between adjacent cells. Chloronema (F) and caulonema (I) after cell dissection and captured chloronemal (G) and caulonemal (J) cells. Bar = 50 μm in all panels.

Morphogenesis of moss gametophytes from protoplasts is similar to morphological development following germination of spores, but the similarity in gene expression level is unknown. This is in contrast to regeneration of the sporophyte from seed plant protoplasts, a process that proceeds through a callus phase and is often difficult to achieve in the laboratory [17]. In the present study, for *P. patens *a new cell wall was regenerated and a polar axis was established within 2 days of generating protoplasts. After 3 days of culture, chloronema containing two to three cells were present (Figure 1A). By day 14, small plants were present, containing both chloronema and caulonema (Figure 1B). In day 14 plants, chloronemal cells were about twice as numerous as caulonemal cells (Table 1). Differentiation of cells into these two cell types is supported by the significant differences in length and chloroplast number per cell (Table 1). By 24 days after protoplast formation, large numbers of buds had formed from protonemal filaments, which represent initiation of leafy shoots (Figure 1C). After one month, mature gametophores were present (Figure 1D).

**Table 1:** Morphological comparison of chloronema and caulonema at day 14.

Trait	Chloronema	Caulonema
Cell no. per plantlet	14.9 ± 3.4	7.7 ± 2.4
Chloroplast no. per cell	49 ± 7	27 ± 6
Cell length (μm)	80 ± 7.0	110 ± 7

Data are mean ± SD. Number of chloronema or caulonema cells per plantlet was scored in approximately 35 plantlets. Number of chloroplasts per cell and cell length were counted in at least 50 chloronema or caulonema cells.

To analyze the gene expression profiles during gametophyte development, we harvested gametophytes at 3, 14, 24, and 30 days after protoplast isolation. To avoid circadian effects, harvesting was conducted at the same time of day. Additionally, 14-day-old live protonemata were used to obtain chloronemal and caulonemal tissues by means of laser microdissection (Figure 1E-J). Each tissue sample comprised mitotically active cells (apical cells and subapical cells generating a side branch; ~20%) and mitotically inactive cells (~80%).

### Laser microdissection and RNA quality

To obtain reliable DGEP results, laser microdissection must produce a sufficient amount of RNA of high integrity. Laser microdissection conditions were optimized for the chloronemal and caulonemal cells of 14-day-old live protonema. The power and duration of laser pulses, especially of the ultraviolet (UV) laser, affect RNA integrity and are dependent on thickness of tissues. We tested the UV laser settings by applying test pulses different intensity and duration. Finally, double the default UV capture power was used. The laser cutting properties had the following parameters: spacing = 700, number of tabs = 2, size = 2.

Of the conditions tested, UV cutting damage was minimized. As shown in Table 2, three sets of chloronemal or caulonemal tissue, respectively, were obtained. Each tissue type contained more than 10,000 cells and each set yielded more than 500 ng RNA, corresponding to >20 pg RNA per cell. In total, 2419 μg and 2555.5 μg RNA were obtained from chloronemal and caulonemal cell populations, respectively. RNA integrity numbers were greater than 7.5.

**Table 2:** Yields of RNA and number of captured cells from chloronemal or caulonemal tissues from 14-day-old protonema of *P. patens*.

Experiment	Cell source	Estimated no. of captured cells	RNA yield (ng)	RNA per cell (pg)
1	Chloronema	3920	896.8	22.9
	
	Caulonema	4540	1019.6	22.5

2	Chloronema	3600	725.8	20.2
	
	Caulonema	4020	960.8	23.9

3	Chloronema	3660	796.4	21.8
	
	Caulonema	2600	575.1	22.1

Total	Chloronema	11180	2419	21.6
	
	Caulonema	11160	2555.5	22.9

### Global gene expression profiles during gametophyte development

Approximately 3.5 to 8.4 million 21-nt cDNA tags were generated using the cDNA library derived from each sample (Additional file 1). Distinct clean tags from each sample were used as data sets for mapping and further analysis (Additional file 2). The full transcriptomic data set was deposited in the GEO database (accession no. GSE33279.

**Figure 2:**
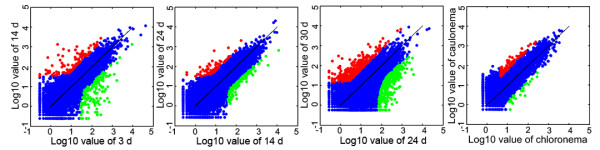
**Gene expression profile analysis during gametophyte development**. The signal intensities of each feature of the DGEP plotted on a logarithmic scale. Red symbols indicate up-regulated genes; green symbols indicate down-regulated genes. Statistical criteria for designation of genes as up- or down-regulated are outlined in the Methods.

We used *edgeR *(*empirical analysis of digital gene expression in R*), a software package available from Bioconductor [18], to normalize for tag distribution per library and determine significance values for differentially expressed genes. The *edgeR *algorithm uses an empirical Bayes analysis to improve power in small sample sizes [19-21]. This accounts for biological and technical variation and has been implemented for tag-based data sets where small numbers of replicates are tested and standard errors disperse further from the mean at low versus high levels of expression [20,22]. We identified 4333 differentially expressed genes during gametophyte development in *P. patens *with false discovery rate (FDR) corrected *P*-values of less than 0.01 (Figure 2; Additional files 3 and 4).

**Figure 3:**
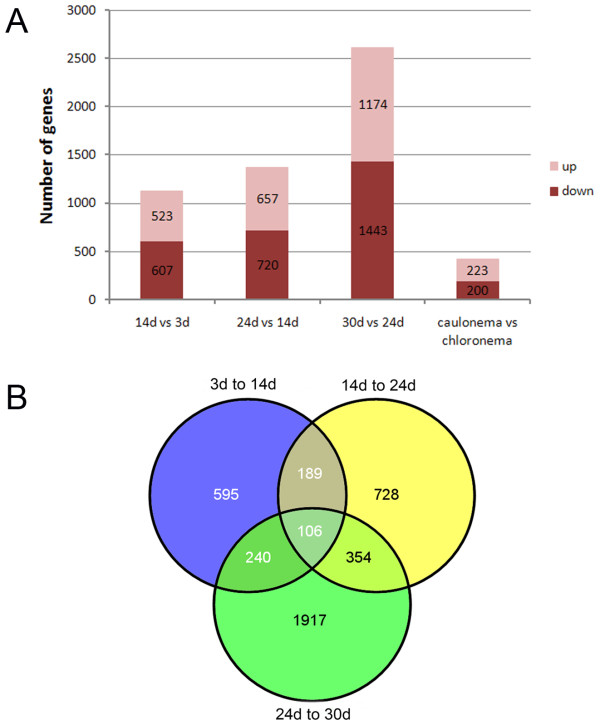
**Genes differentially expressed at different developmental stages or in individual tissue types of protonema during gametophyte development**. Genes differentially expressed during specific developmental phases of gametophyte development and tissue type-specific DEGs in a 14-day old protonema (A) were separated into two groups according to whether they were significantly up-regulated or down-regulated. Venn diagrams (B) showing the number of differentially expressed genes during specific developmental stages of gametophyte development.

To ensure that meaningful changes were considered, we applied stringent statistical criteria to select genes, as described in the methods. A total of 4333 genes were identified as differentially expressed by tag-based DGEP (Figure 2). Of these genes, 4129 were differentially expressed as a function of developmental stage (Additional file 4) and 423 were expressed differentially based on tissue type (i.e., chloronemal tissue versus caulonemal tissue) (Figure 3; Additional file 4). Among the former 4129 genes, 3240 genes were preferentially expressed at a single developmental stage, 783 were expressed preferentially at two developmental stages, and 106 at more than two stages (Figure 3B). Of the 423 genes differentially expressed in the two cell types, 223 were preferentially expressed in caulonemal cells (Figure 3A; Additional file 4).

The top 15 significantly enriched gene ontology (GO) terms for differentially expressed genes (DEGs) (*p *< 0.01) are listed in Additional file 5. From 3 days to 14 days, biological processes of enriched DEGs focused on generation of precursor metabolites and energy, organic substance metabolic processes, energy reserve metabolic processes, regulation of photosynthesis light reaction, and regulation of generation of precursor metabolites and energy. The molecular functions of enriched DEGs were oxidoreductase activity (acting on the CH-CH group of donors, with NAD or NADP as acceptor) and sugar transmembrane transporter activity. However, the cellular components were organellar large or small ribosomal subunit, proton-transporting two-sector ATPase complex, and membrane.

During bud formation (14 days to 24 days), significant enriched DEGs differed from those detected during protonema development. The biological processes mainly focused on cellular component biogenesis at cellular level and the photosynthetic electron transport chain, as well as generation of precursor metabolites and energy. The molecular functions were similar to those detected after 3 days to 14 days. However, the cellular components were focused on the photosystem and chloroplast envelope.

Other than the stages mentioned above, significant enriched DEGs concentrated on generation of precursor metabolites and energy, and functioned in acid-ammonia (or amide) ligase activity at the leafy shoot development stage (Additional file 5).

For tissue-specific DEGs, significant enriched GO terms were mainly involved in processes of cell wall component metabolism, modified amino acid metabolism, ATP biosynthesis and metabolism, and oxidation-reduction, and functioned in transporter activities, such as hydrogen ion, inorganic cation, and substrate-specific transmembrane transport. These processes and molecular functions were mainly implemented in the proton-transporting two-sector ATPase complex, intracellular organelle, ribosomal subunit and plastids.

### Validation of DGEP data by quantitative real-time PCR

**Figure 4:**
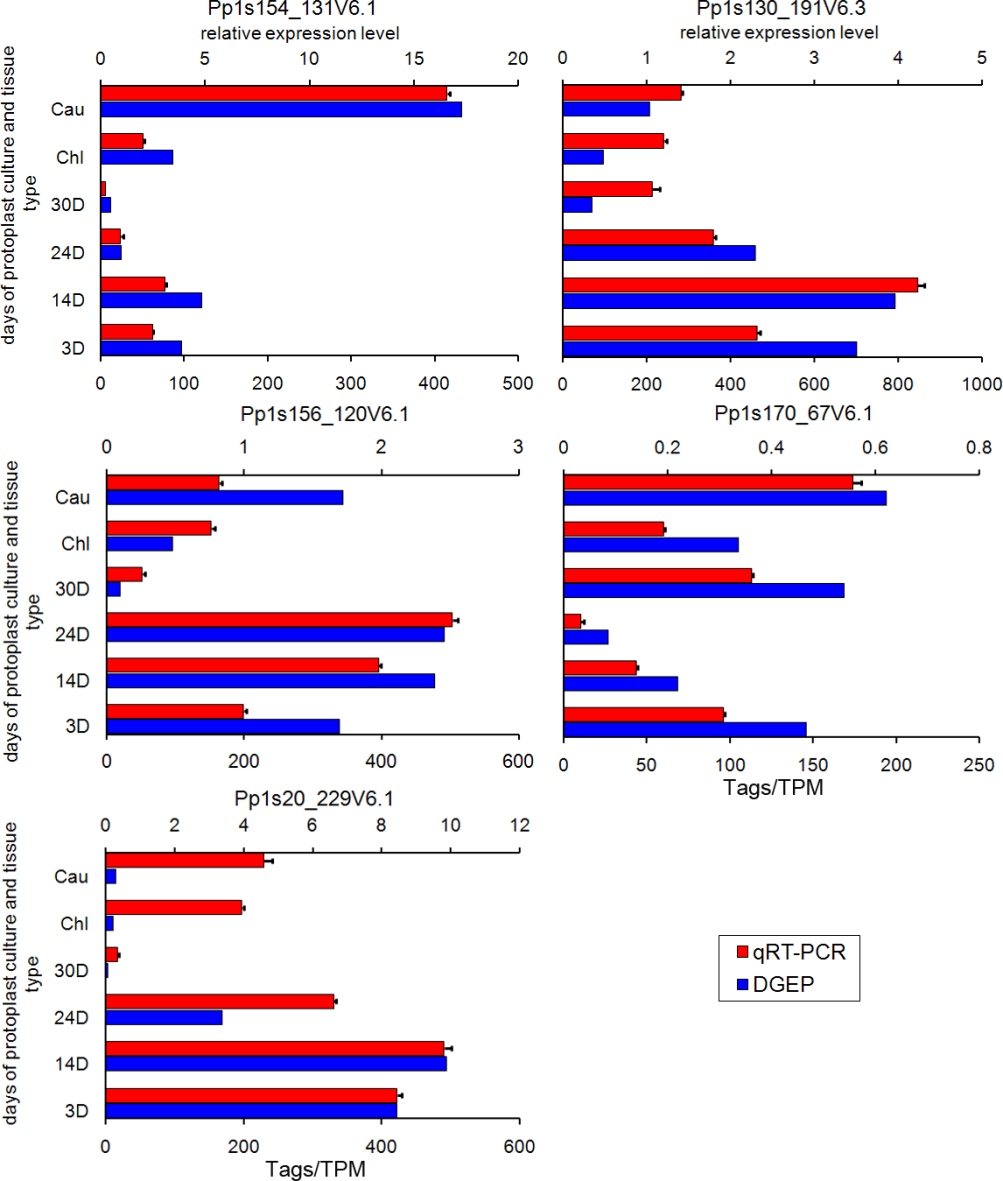
**Gigital gene expression tag profiling and quantitative real-time PCR analysis of the expression of five randomly selected genes**. All real-time PCR reactions were repeated three times and the data are presented as the mean ± SD. The *x*-axis indicates the sampling time-points and cell types. The *y*-axis shows the expression levels: the left shows tag number per million tags by DGEP and the right shows the relative expression level by qRT-PCR.

To validate the accuracy and reproducibility of the DGEP results, the transcriptional levels of five randomly selected genes were measured by quantitative real-time PCR (qRT-PCR), in comparison with two internal genes that showed relatively stable expression levels (Additional files 6 and 7). In all cases, the trends of real-time PCR-based expression profiles among these genes were similar to those obtained by DGEP analysis (Figure 4).

### Developmental stage-specific profiling in the *P. patens *gametophyte

**Figure 5:**
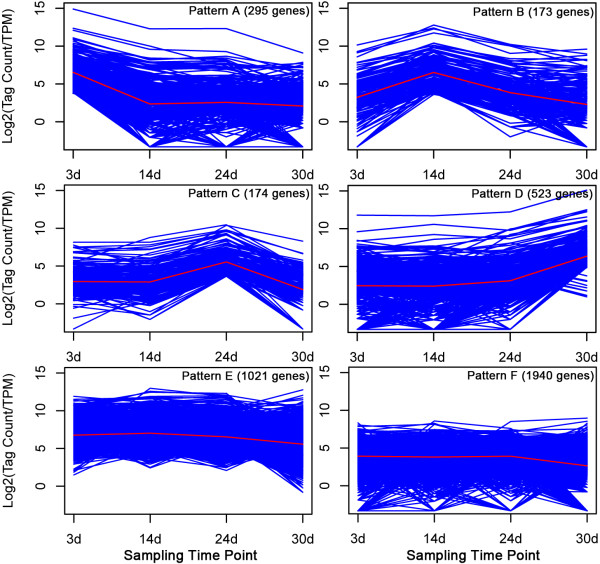
**Patterns of gene expression by K-means cluster analysis in the developing gametophyte of *P. patens***. Differentially expressed genes across all four time-points were grouped into six clusters using the K-means clustering algorithm. The *y*-axis gives the tag count (on a log scale) of differentially expressed genes. Each line represents a different gene.

To elucidate the developmental stage-specific gene expression profiles, we further analyzed the expression patterns of all differentially displayed genes, with the expression level at 3 days taken as a common reference. Based on this analysis, the 4129 differentially expressed genes were grouped into six patterns using a K-means clustering algorithm (Figure 5; Additional file 8). Pattern 1 genes were highly expressed at day 3 and decreased to lower levels at subsequent stages of development; in contrast, expression of pattern 4 genes was low throughout development until day 30 when their expression rose. Expression of pattern 2 genes peaked at day 14, that of pattern 3 genes peaked at day 24, and pattern 6 genes showed broad optimal expression that spanned from days 14 to 24.

After 3 days of development, cell walls had regenerated and chloronema had formed (Figure 1A). Among the annotated pattern 1 genes (i.e., those with maximal expression at day 3), most were related to material and energy metabolism, and mainly participated in carbon fixation, protein and amino acid metabolism, lipid metabolism, and starch and sucrose metabolism (Additional file 8). In addition, this group contained several genes related to transport and ion binding, such as the auxin polar transport-related genes *ABCB4 *(Pp1s391_45V6.1|PACid:18056701) and *ABCB19 *(Pp1s252_67V6.1|PACid:18037761). Pattern 1 also included several transcription factors and other regulatory genes. For example, this group included genes that encode a putative MADA-box family member (*PHE1*, Pp1s7_353V6.1|PACid:18040126), a WRKY transcription factor (*WRKY42*, Pp1s158_166V6.1|PACid:18042126), a cytokinin response factor (*CRF2*, Pp1s7_481V6.1|PACid:18051877), two putative MYB family members (Pp1s66_90V6.1|PACid:18038312; Pp1s2_312V6.1|PACid:18052823), a bZIP family member (*BZO2H3*, Pp1s213_13V6.2|PACid:18049359) and a homologue of a gene involved in chromatin remodeling (*CHR4*, Pp1s223_99V6.1|PACid:18036570). Moreover, several protein kinase-related genes were also preferentially expressed. Finally, pattern 1 genes included a few putative stress-responsive genes, such as heat shock protein 70 members, peroxidase/catalase superfamily members, and a putative gene encoding a CDC48 protein (*CDC48*, Pp1s296_11V6.2|PACid:18068409). However, it is not clear whether or not expression of these stress-responsive genes reflects the inherent developmental program.

Pattern 2 genes were expressed maximally at 14 days, which represented an early stage of gametophyte development with vigorous growth (Figure 1B). Not surprisingly, compared to day 3, genes involved in ribosomal protein synthesis installation represent a major proportion of the total, which mainly functions in plastids (Additional file 8). Genes related to cell wall biosynthesis, light capture and carbon assimilation continued to represent a major component. With regard to regulatory proteins, genes encoding a calmodulin showed significant expression changes (*CAM5*, Pp1s40_127V6.1|PACid:18070060). Moreover, a putative gene involved in histone lysine methylation (*ATX1*, Pp1s67_56V6.1|PACid: 18050867) also showed peak expression.

Genes in pattern 3 were expressed maximally at day 24, a stage in which the leafy shoot initials form and which represents the transition from the two-dimensional protonemal network to the three-dimensional adult gametophyte (Figure 1C). At this stage, although genes involved in material and energy metabolism were still the most abundant classes, their dominance decreased and the representation of genes among all other categories increased, including a few putative transcription factors (*SHP1*, Pp1s267_56V6.1|PACid:18072078; HB-8, Pp1s188_95V6.1|PACid:18072307; *GATA19*, Pp1s121_36V6.1|PACid:18055435; *HY5*, Pp1s80_72V6.1|PACid:18053259), protein kinase (*BAM2*, Pp1s59_314V6.1|PACid:18060049; *SK13*, Pp1s52_55V6.1|PACid:18074399; *VIK*, Pp1s136_112V6.1|PACid:18036754) and methyltransferase categories (*ACL5*, Pp1s41_50V6.2|PACid:18058199; *STE14B*, Pp1s112_210V6.2|PACid:18064877) (Additional file 8). Interestingly, one of the pattern 3 genes was a putative transcription factor HB-8, a putative homologue of which has been characterized in Arabidopsis and is involved in the differentiation of preprocambial cells into xylem during leaf vein formation [23]. Because bryophytes lack a vascular cambium, these results suggest that this transcription factor originally had a role in the development and the evolution of a pervasive multicellular plant body.

Genes in pattern 4 were specifically expressed at the adult leafy shoot stage (Figure 1D). At this stage, the proportion of metabolic genes increased to almost half and the proportion of protein synthesis genes decreased to a similar level to that of all other categories (Additional file 8). Approximately 40 members of several transcription factor families significantly increased in transcript level, such as members from the zinc finger, bHLH, MYB, BAH, bZIP, CHASE and PLATE superfamilies. More than 20 protein kinase-encoding genes were also preferentially expressed. Interesting regulatory genes in this group included those encoding a putative ethylene response gene (*ETR1/EIN1*, Pp1s115_107V6.1|PACid:18060143) a putative thylene insensitive 3 (*EIN3*, Pp1s11_337V6.1|PACid:18048657), an auxin response factor 6 (*ARF6*, Pp1s133_56V6.1|PACid:18061618), a growth-regulating factor 2 (*GRF2*, Pp1s344_39V6.1|PACid:18068535), a putative mitogen- activated protein kinase 12 (*MPK12*, Pp1s20_265V6.1|PACid:18057264), a putative CHASE domain-containing histidine kinase protein (*WOL*, Pp1s50_141V6.1|PACid:18059272) a histone lysine methylation-related gene (*VIP3*, Pp1s22_260V6.1|PACid:18042729), and a putative S phase-associated protein 1 (*SKP1*, Pp1s7_342V6.1|PACid:18051944).

### Candidate genes involved in protonema differentiation

Vegetative development of *P. patens *involved only a few cell types. In our experimental conditions, a protonema initially comprised only chloronemal cells within the first 10 days. Subsequently, the chloronemal tip cells began to change, ultimately differentiating into caulonemal cells. By day 14, the protonemal colony included well-developed chloronema and caulonema (Figure 1B). To understand the molecular basis that leads to the differences between the two cell types, we isolated the two cell types by laser-capture microdissection and used DGEP to profile gene expression. In total, we identified 423 DEGs (Figure 3A). Of these, 200 DEGs were preferentially expressed in chloronemal cells and 223 in caulonemal cells.

Of those genes preferentially expressed in chloronemal tissue, the majority functioned in metabolism and protein synthesis (Additional file 4). Additionally, a putative zinc finger (DNL type) transcription factor and three protein modification-related genes were specifically expressed. Among the 223 genes preferentially expressed in chloronemal tissue, significantly enriched genes functioned in ion binding, protein binding, RNA binding and structural molecule actchloronemaity (Additional files 4 and 5). Compared with caulonemal tissue, genes preferentially expressed in chloronemal tissue represented four major categories: organic substance and energy metabolism (especially photosynthesis), protein synthesis and proteolysis, transport, and cell wall synthesis and signaling pathways (Additional files 4 and 5). Genes related to several transcription factors in the two tissues (*ABI3*, Pp1s173_143V6.1|PACid: 18050971; *BTF3B*, Pp1s29_196V6.1|PACid: 18048872 & Pp1s470_10V6.2|PACid: 18073306; *CDF3*, Pp1s69_11v6.1|PACid: 18049422; *PIF3*, Pp1s84_22V6.1|PACid: 18054521 & Pp1s147_126V6.1|PACid: 18053936) may play key roles during the differentiation of chloronemal and caulonemal tissues and were chosen as candidates for discussion and future study.

## Discussion

### Combination of laser-capture microdissection and DGEP for tissue-specific transcript profiling-digital *in situ *hybridization

Plant development reflects both endogenous genetic programs and responses to exogenous events [7]. Analyses of global gene expression can reveal much about how genes function and how their products interact during development. Most previous gene expression analyses sampled whole organs or tissues [7]. Although these studies have provided valuable information, they are limited by the composite nature of plant organs, which consist of multiple tissues. We expect each cell type to have a unique transcriptome [24]. A transcriptomic analysis of a complete organ provides gene expression information integrated over all cell types, and is thus not particularly informative about cellular differentiation.

Technical advances have made it possible to study global patterns of gene expression in an individual tissue, which increases the information revealed by expression profiling. A transcriptome from a single tissue can now be obtained by using laser-capture microdissection, an approach whereby a cell is physically isolated from the surrounding tissue [25]. Several research groups have used laser-capture microdissection successfully to isolate specific plant cells [26-29]. Typical laser capture microdissection is used on sectioned material, but the two-dimensional nature of the protonemal growth habit of *P. patens *allowed us to capture cells from living material, thereby minimizing undesirable changes in gene expression during sample preparation. Because of the difficulty of obtaining more than 10,000 apical or subapical cells of chloronemal and caulonemal tissues, we captured chloronemal and caulonemal samples that contained both mitotically active cells and mitotically inactive cells for DGEP analysis.

DGEP is conducted on a Genome Analyzer 2x system using an eight-lane flow cell, which can generate 90 to 100 million reads per run (http://www.Illumina.com). In the present study, we successfully profiled chloronemal and caulonemal gene expression in *P. patens *by combined laser-capture microdissection and DGEP. The results demonstrated LCM-mediated DGEP analyses can be used to conduct global profiling of gene expression on individual tissues captured from live plants. We report here LCM-based methods to isolate chloronemal and caulonemal tissues from live protonema free of detectable contamination with intact non-target cells in *P. patens *(Figure 1). This is the first report of application of an LCM-based method to capture individual tissues from live plants. In this study, 358 genes were preferentially expressed in chloronemal or caulonemal tissue. Our data provide important clues for elucidation of the molecular mechanism of cell differentiation and provide a useful baseline for future digital gene expression mapping.

### Altered metabolism during *P. patens *gametophyte development

At day 3, half of the preferentially expressed genes (i.e., pattern 1 genes) functioned in metabolism (Additional files 4 and 5), most of which were involved in the citrate cycle, pentose phosphate pathway, and metabolism of pyruvate, starch, and sucrose. Although earlier stages were not sampled, these findings suggest the regeneration process requires considerable metabolic reprogramming. It would be interesting to determine whether a similar pattern emerged in the first few days following spore germination.

In our culture conditions, protonema containing two cell types emerged between days 3 to 14 (Figure 1). Not surprisingly, transcripts encoding genes involved in photosynthesis and protein synthesis represented the bulk of the genes preferentially expressed at day 14. Between days 14 and 24, the plants transition from a two-dimensional protonemal network to three-dimensional structure containing leafy shoots, although by day 24 the leafy shoots were mostly still buds. Along with this development, many new metabolism-related genes were expressed, which implied distinct metabolic pathways operate during this phase of gametophyte development.

By day 30, a mature three-dimensional gametophyte consisting of leafy shoots and rhizoids was attained. Interestingly, at this stage, a gene encoding a homologue of thale cress *ROOTHAIR DEFECTIVE 3 *(*RHD3*, Pp1s33_217V6.1|PACid:18056995) was preferentially expressed. In Arabidopsis, *RHD3 *encodes an evolutionarily conserved protein with GTP-binding motifs that is required for expansion of both roots and root hairs, and is implicated in the control of vesicle trafficking between the endoplasmic reticulum and the Golgi compartments [30,31].

A previous study by Menand et al. [32] suggested *ROOT HAIR DEFECTIVE 6 *(*RHD6*), a homologue of *RHD3*, controls root hair development in Arabidopsis and rhizoid development in *P. patens*. This result supports the hypothesis that early land plants were bryophyte-like and possessed a dominant gametophyte, and that later the sporophytes rose to dominance. Furthermore, this finding suggests the increase in morphological complexity of the sporophyte body in the Paleozoic resulted at least in part from the recruitment of regulatory genes from gametophyte to sporophyte.

Comparison of the two protonemal cell types showed a number of genes related to protein synthetic installation, cell wall metabolism, photosynthesis and transport were specifically expressed in chloronema. This cell type forms initially and the more vigorous caulonema differentiate subsequently. The expression pattern of chloronema might indicate the primary function of these cells is to give rise to the caulonemal cells and it is the latter that take on diverse physiological functions and lead to the continued development of the gametophyte; in addition, accumulation of transcript products in chloronemal cells might contribute material and energy for caulonemal cell differentiation.

### Cell division genes

The gametophyte progresses from two or three cells at day 3 to tens of thousands of cells by day 30. Not surprisingly, many genes related to control of cell division were differentially expressed, among which were the following genes.

Proteolysis is considered to be important steps in ensuring unidirectional progression of the cell cycle by triggering rapid degradation of target proteins that in turn drive the cycle forward [33]. The SCF complex, consisting of SKP1, Cullin, and F-box, could associate with E3 ligase and help to recruit and degrade the target proteins during the G1-S transition of the cell cycle [34]. In the present study, genes encoding SKP1, Cullin 1 and Cullin 4 were preferentially expressed after 30 days, which suggests a role for the SCF complex pathway in the regulation of cell division in leafy shoots during gametophyte development in *P. patens*. The auxin response also depended on regulation of the SCF E3-ubiquitin-protein complex [35]; consequently, increased SKP1 transcription might promote an endogenous auxin response and activate the cell division cycle.

### Expression of genes related to hormone signaling during gametophyte development

Hormones regulate plant development by a complex signal response network. Similar to higher plants, growth and development in *P. patens *is regulated both by environmental factors and phytohormones [36,37]. Genes involved in the signaling pathway of three major plant hormones, namely auxin, ethylene and cytokinins, are discussed in this section. In the present study, eight preferential DEGs involved in the auxin signaling pathway were expressed from days 3 to 30 (Additional file 8). Of these, two genes that belong to the family of ATP-binding cassette (ABC) transporters, *ABCB4 *and *ABCB19*, were preferentially expressed at day 3. In thale cress, the ABCB4 and ABCB19 proteins were required for polar transport of auxin (indole-3-acetic acid) [38,39]. In addition, the ABCB19 protein possibly regulated auxin-dependent responses by influencing basipetal auxin transport in the root and influenced the auxin transport process that acts through cry1 or phyB to control hypocotyl growth during de-etiolation in seedlings [40]. Auxin transport is mediated at the cellular level by three independent mechanisms that are characterized by the PIN-formed (PIN), P-glycoprotein (ABCB/PGP), and AUX/LAX transport proteins [41]. PIN and ABCB transport proteins, which are best represented by PIN1 and ABCB19 (PGP19), coordinately regulate auxin efflux. When PIN1 and ABCB19 coincide on the plasma membrane, their interaction enhances the rate and specificity of auxin efflux, and the dynamic cycling of PIN1 is reduced. This finding suggests ABCB19 stabilizes PIN1 localization at the plasma membrane in discrete cellular subdomains where PIN1 and ABCB19 expression overlaps [41,42].

Another important auxin response gene was *auxin response factor 6 *(*ARF6*), of which one copy (Pp1s133_56V6.1|PACid:18061618) showed maximal expression at day 30 (Additional file 8). The other copy (Pp1s316_22V6.1|PACid:18051030) showed peak expression in caulonema (Additional file 5). The *ART6 *gene, as a positive regulator, is involved in the crosstalk of auxin and light signaling pathways and auxin homeostasis and controls adventitious root initiation in Arabidopsis [43]. A previous study suggests auxin promotes the development of caulonema cells in *P. patens *[44]. Our results demonstrate auxin promotes growth and differentiation of caulonema and three-dimensional leafy shoots.

Ethylene is a plant hormone that regulates many processes, such as seed germination, root hair development, fruit ripening and stress responses, in higher plants [45]. According to the model elaborated for Arabidopsis [46], ethylene is perceived by specific receptors that act as negative regulators of the ethylene response. Five ethylene receptors, i.e., ETR1/EIN1, ERS1, EIN4, ETR2, and ERS2, are present in thale cress and dominant negative mutants of each that confer ethylene insensitivity are reported [47]. The *P. patens *genome codes for six putative ETR-like ethylene receptors and two putative 1-aminocyclopropane-1-carboxylate (ACC) synthases as well as two transcription factors, although there is no clear evidence that *P. patens *responds to ethylene [9]. We found a putative ethylene receptor (ETR1/EIN1) was preferentially expressed at day 30 and a putative ethylene insensitive gene (*EIN3*) accumulated at day 30. In Arabidopis, EIN3 functions as a nuclear transcription factor that initiates downstream transcriptional cascades for ethylene responses, including seedling de-etiolation, modulation of nitric oxide-related iron acquisition and homeostasis, and plant innate immunity [48-51]. Therefore, we speculate the ethylene signaling pathway might also play a role during *P. patens *gametophyte development.

Some reports suggest cytokinins are also important phytohormones involved in *P. patens *development, which can induce bud formation in mosses in a concentration-dependent manner [52]. In the present study only two genes involved in the cytokinin signaling pathway were identified. A putative cytokinin response factor 2 (*CRF2*) and a putative CHASE domain-containing histidine kinase protein (*WOL*) showed maximal expression at day 3 and day 30, respectively (Additional file 8). These data provide clues to the molecular mechanism of developmental processes regulated by phytohormones in *P. patens*.

### Transcription factors

During protoplast regeneration into a gametophyte, more than 80 transcription factors show changed expression levels (Additional file 4). Among the differentially expressed transcription factors, several were preferentially expressed at day 3 (Additional file 8). One of these encodes a putative BZO2H3 homologue, which belongs to the bZIP transcription factor family. Recently, BZO2H3 was reported to be a sensitive integrator of transient abscisic acid and glucose signals in Arabidopsis [53]. Another differentially expressed transcription factor is WRKY42, a putative homologue of the WRKY transcription factor family (Group II-b) in Arabidopsis. The WRKY proteins are a superfamily of transcription factors with up to 100 representatives in Arabidopsis. Family members appear to be involved in the regulation of a variety of physiological processes unique to plants, including pathogen defense, senescence and trichome development [54]. We suggest, therefore, that these two transcription factors play a role in protoplast regeneration.

One transcription factor that showed peak expression on day 24 is a putative homologue of class III homeodomain-leucine zipper (HD-ZIP III) transcription factors, HB-8. In thale cress, HB-8 binds to the promoters of genes predominantly expressed in vascular tissues [55], and acts as a transcription factor in vascular meristems to promote proliferation of procambial cells and suppress their differentiation into vascular cells [23]. Promoting proliferation while suppressing differentiation might be necessary to form the leafy gametophyte shoot on a physiologically appropriate time scale.

At day 30, one of the preferentially expressed transcription factors was a member of the GRF gene family, which comprises nine members including growth-regulating factor 2 (GRF2; Additional file 8). In Arabidopsis, overexpression of AtGRF1 and AtGRF2 resulted in larger leaves and cotyledons, as well as in delayed bolting of the inflorescence stem when compared to wild-type plants [56]. It is possible that these transcription factors play similar roles in *P. patens*.

### Epigenetic modification and gametophyte development

Protein methylation is one type of post-translational modification. Histones that are methylated on certain residues can act epigenetically to repress or activate gene expression [57,58]. In the plant kingdom, protein methylation plays a fundamental role in the regulation of diverse developmental processes [59]. In the present study, two putative protein methylation-associated genes showed differential expression during protoplast regeneration into a gametophyte (Additional file 8). Of these genes, STE14B was preferentially expressed at day 24 and encodes a putative homologue of the isoprenylcysteine carboxyl methyltransferase family, which is involved in post-translational processing of proteins with a C-terminal CaaX box [60]. A putative VIP3 homologue exhibited maximal expression at day 30. This gene is predicted to encode a transducin/WD40 repeat-like superfamily protein with a DWD motif, which is involved in protein complex formation. In Arabidopsis, this gene is involved in the timing of flowering and flower development. Loss-of-function of this gene leads to a redistribution of H3K4me3 and K3K36me2 modifications within genes but not a change in the overall abundance of these modifications within chromatin [61,62]. These expression results suggest epigenetic modification of proteins play important roles during gametophyte development in *P. patens*. Further research is still needed to uncover the molecular mechanisms that control gametophyte development.

## Conclusions

We analyzed the transcriptome during the transition between sequential developmental stages, and as cells differentiate, during gametophyte development of *P. patens*. The transcript levels of 4333 genes were significantly increased or reduced, of which the majority changed only during a specific developmental stage or in an individual tissue type. Our results provide an extensive catalogue of regulatory factors and related genes involved in cell division, growth, and differentiation during gametophyte development in *P. patens. *Potential applications of these data include identification of candidate genes for evolutionary developmental studies, as targets for reverse genetic studies of plant development, and as tools for cell-by-cell mapping of genes involved in plant development.

## Methods

### Protoplast isolation and culture

Protonemal tissues of *Physcomitrella patens *subsp. *patens *were subcultured at weekly intervals on the surface of cellophane that overlayed solid BCD medium containing 5 mM ammonium tartrate and 1% glucose (BCDAG) [63]. Protoplasts were isolated from protonemata according to a modified protocol of Rother et al. [64]. Ten Petri dishes of six-day-old protonemal tissues were collected and placed in 10 mL of 0.5% driselase (Sigma-Aldrich, Taufkirchen, Germany) in 8% (w/v) mannitol. After 30 min agitation in the dark, the cells were passed successively through sieves with a mesh size of 100 μm and 50 μm. The protoplast suspension was allowed to stand for 15 min, and then was centrifuged for 5 min at 600 rpm. The pellets were washed with 8% mannitol and the number of protoplasts was counted in a hemocytometer after a second centrifugation. The freshly isolated protoplasts were transferred to 9 cm Petri dishes containing BCDAG medium and cultured in the dark for 24 hours at 25 ± 1°C. The cultures were incubated under light intensity of 55 μmol m^-2 ^s^-^^1 ^under a 16/8 hour (light/dark) photoperiod at a constant temperature (25°C).

### Laser-capture microdissection

Cell type-specific gene expression profiling was performed on 14-day-old protonema using a laser-capture microdissection system (Arcturus^XT^, Arcturus, Mountain View, CA, USA) to isolate chloronemal and caulonemal cells separately. Live protonema was placed on the 'flat' side of the framed membrane slide coated with poly-L-lysine, and mounted on a regular glass slide. Immediately thereafter, cells were isolated according to the manufacturer's instructions (http://www.bmbio.com/file/201734.pdf). Each slide was handled for less than 30 min to ensure cell viability. Cell sets of more than 10,000 cells of each cell type were captured and used for RNA isolation.

### Total RNA preparation and quality control

To generate developmental stage-specific mRNA expression profiles, total RNA was isolated with TRIzol reagent (Invitrogen, Carlsbad, CA, USA) from 100 mg samples, including 3 day to 30 day. RNase-free DNase (RQ1; Promega Corporation, Madison, Wisconsin, USA) was used to remove genomic DNA. RNA from microdissected chloronemal or caulonemal tissues was extracted from the cells on the CapSure Macro LCM Cap (CapSure Macro; Arcturus) using the PicoPure RNA Isolation Kit (Arcturus) according to the manufacturer's protocol. The CapSure Macro LCM Cap with captured cells was inserted onto a microcentrifuge tube (Applied BioSystems) containing 50 μL extraction buffer. After incubation for 30 min at 42°C, the assembly was centrifuged at 800 × *g *for 2 min to collect the cell extract in the microcentrifuge tube. RNA purification was conducted using the RNA purification column with conditioning buffer and 70% ethanol. The RNA was washed with elution buffer.

The yield of RNA was determined by measuring the absorption at 260 nm with a NanoDrop 1000 spectrophotometer (Thermo Fisher Scientific Inc., Marietta, OH, USA). Integrity of the RNA was evaluated on an Agilent 2100 Bioanalyzer, using an RNA 6000 LabChip kit (Agilent Technologies, Palo Alto, CA). Only RNA with RNA integrity numbers greater than 7.5 were used for DGEP and qRT-PCR analysis. For each tissue type and sampling time-point, three biological replicates were combined for DGEP or separated for further expression analysis by qRT-PCR.

### Digital gene expression tag profiling and data analysis

To obtain gene expression profiles, sequencing libraries containing 21-nt tags were prepared from 6 μg total RNA (for developmental stage-specific expression profiles) or 2 μg total RNA (for cell type-specific expression profiles) using the Illumina Gene Expression Sample Prep Kit (Illumina, San Diego, CA, USA). Oligo (dT) magnetic beads were used to adsorb and purify mRNA, and then to guide reverse transcription to synthesize double-stranded cDNA. While on the beads, double-stranded cDNA was digested with an anchoring restriction endonuclease *Nla*III that recognizes and cuts off the CATG sites on the cDNA, and ligates to an Illumina-specific adapter A. The junction of the Illumina adapter A and CATG site is the recognition site of *Mme*I, which cuts the cDNA at 17 bp downstream of the CATG site.

Following *Mme*I digestion and dephosphorylation with shrimp alkaline phosphatase (USB Corporation), the cDNAs were purified and a second Illumina adapter B was introduced at the 3' end of the tags, which generated tags with different adapters at both ends to form tag libraries. After 15 cycles of linear PCR amplification, 85 base strips were purified by 6% TBE PAGE electrophoresis. The DNA quality was assessed and quantified using an Agilent DNA 1000 series II assay (Agilent) and the DNA sample was diluted to 10 nM. The strips were then denatured, and the single-chain molecules were fixed onto the Solexa Sequencing Chip (flowcell). Cluster generation and sequencing was performed on the Illumina cluster station and Genome Analyzer system (Illumina, version 1.0) following the manufacturer's instructions. Each tunnel generated millions of raw reads with a sequence length of 35 bp (target tags plus 3' adaptor). Each molecule in the library represented a single tag derived from a single transcript.

Raw sequences were extracted from the resulting image files using the open source Firecrest and Bustard applications (Illumina). Raw reads were transformed into clean tags by filtering out adaptor-only tags, low-quality tags (containing ambiguous bases), tags that were too long (>21 nt) or too short (<21 nt), and those present in a single copy only and hence assumed to represent sequencing errors. Using blastn searches, comparison of the sequences was carried out using the JGI *Physcomitrella patens *genome database (ftp://ftp.jgi-psf.org/pub/JGI_data/phytozome/v7.0/Ppatens/annotation/) and National Center for Biotechnological Information databases [65]. All clean tags were mapped to *P. patens *reference sequences. For conservative and precise annotation, only sequences with a perfect match or 1-nt mismatch were considered further. Clean tags mapped to reference sequences from multiple genes were filtered. The remainder of the clean tags were designed as distinct clean tags. The number of distinct clean tags for each gene was calculated and then normalized to the number of transcripts per million clean tags (which is a standardized indicator of the transcript copies in every 1 million clean tags) [66,67]. The saturation analysis was performed to check whether the number of detected genes continues to increase when the sequencing amount (total tag number) increases. Sequences were manually assigned to functional categories based on the analysis of scientific literature.

Only a small portion of the *P. patens *genome has been studied. Therefore, for those genes whose functions could not be inferred from the *P. patens *genome, a homology search in The Arabidopsis Information Resource (TAIR) 10 database (http://www.arabidopsis.org/) was conducted and the annotation of homologues in *A. thaliana *was used.

A rigorous algorithm to identify differentially expressed genes between two samples was developed for significance testing [68]. The *P*-value corresponds to the differential gene expression test. The FDR was applied to determine the threshold *P*-value in multiple tests through manipulation of the FDR value [69].

The number of tags mapped to a given gene was considered to represent the expression level of this gene. Expression levels of a gene from two distinct samples were compared to give an expression difference. We classified the gene as differentially expressed only when the expression difference was more than two-fold with *P*-value < 0.01 and FDR < 0.001.

To determine the main biological functions, DEGs were mapped to every node of the Gene Ontology (GO) database (http://www.geneontology.org/) and the gene number of each node was calculated with GenMAPP v2.1, a program designed to perform a global analysis of gene expression or genomic data in the context of hundreds of pathway MAPPs and thousands of GO terms (http://www.genmapp.org/download_v2.1.php). GO enrichment analysis of functional significance terms in the GO database was applied (Fisher's test, *p *< 0.01) to map all DEGs to terms in the GO database, looking for significantly enriched GO terms in DEGs compared to the genome background. For cluster analysis of DEGs at different developmental stages, the comparison between two consecutive time-points was transformed into a comparison using the day 3 time-point as a common reference point. The K-means algorithm was used to produce groups of DEGs using the Calculated Means mode with Euclidean distance based on log fold-change data.

### Significance testing

To compare gene expression profiles between two sequential time-point samples, the *edgeR *package (http://www.bioconductor.org/packages/2.3/bioc/html/edgeR.html) was used to adjust for differences in library size and therefore raw read counts per gene (or per tag) were directly used as input. We used a moderated, gene-wise dispersion analysis for all four sample's data sets separately with a weighted parameter (prior.n) of 100. Because our data lacked a biological replication, we applied more than 100 tags for each gene as a cut-off, in order to increase the reliability of the results. The significance threshold for differential expression was *p *< 0.01 after correction using a Benjamini Hochberg FDR of 0.01.

### Quantitative real-time PCR

For each sample, cDNAs were synthesized from 2 μg total RNA (developmental stage-specific expression profile) or 0.5 μg total RNA (cell type-specific expression profile) using the Invitrogen Reverse Transcription Reagents Kit. Gene-specific primers were designed and assessed with commercial software (Primer 5 and Oligo 6, respectively; Applied Biosystems, Foster City, USA) and synthesized by Shanghai Sangon Technical Services (Shanghai, China). Primers used in the qRT-PCR analysis are listed in Additional file 6. The qRT-PCR reactions were performed using a Corbett Research Rotor-Gene 3000 under the following conditions: 94°C for 5 min (one cycle); 94°C for 20 s, 50°C to 60°C for 20 s, and 72°C for 20 s (50 cycles). Transcript abundance was identified using the SYBR Green PCR Master Mix (TaKaRa Bio, Japan). Each reaction contained 1 μM mix buffer, 0.2 μM each primer, and about 2 ng cDNA in a final volume of 25 μL. Three sample replicates were employed for each sample and a template-free negative control was performed. Data were normalized relative to Pp1s40_169V6.1 or Pp1s17_377V6.1, which exhibited relatively stable expression levels in all day-3 to day-30 samples and in chloronema and caulonema samples (Additional file 7). Melting curves were performed on the product to test if only a single product was amplified without primer dimers and other bands. The products with all primer combinations were visualized in a 2% agarose gel to confirm the generation of a single product of the correct size. Relative quantitative analysis was performed using comparative quantitation by Rotor-Gene Real-Time Analysis 6.1 software.

## Authors' contributions

LHX carried out the plant material preparation, RNA extraction, sequence analysis, and contributed to data interpretation and manuscript writing. HW prepared part of the plant material. PW provided assistance with significance testing of the sequence data using *edgeR*. TYK participated in manuscript modification. YKH conceived the study, led the experiment design and coordinated all the research activities, contributed to interpretation of the data, manuscript writing and modification. All authors read and approved the final manuscript.

## Supplementary Material

Additional file 1 **Summary of Solexa tags in the cDNA libraries**. Click here for file

Additional file 2 **Distribution of distinct clean tags in each sample**. Click here for file

Additional file 3 **Smear plots from the *edgeR*-based analysis of gene expression**. Genes are plotted based on their log-fold change of transcript abundance between two compared samples on the *y*-axis and log concentration on the *x*-axis for raw tag libraries separately. Differentially expressed genes are shown in red. Click here for file

Additional file 4 **Complete list of transcripts attributed to different developmental stages and chloronemal or caulonemal tissues in 14-day-old protonema during *P. patens *gametophyte development**. Click here for file

Additional file 5 **Gene ontology enrichment results for differentially expressed genes**. Click here for file

Additional file 6 **The primer sequences of the randomly selected and internal genes used for real-time PCR analysis**. Click here for file

Additional file 7 **Expression level of internal genes in each sample in the real-time PCR analysis**. Click here for file

Additional file 8 **Complete list of each K-means cluster during gametophyte development in *P. patens***. Click here for file
