# Supplementary figures and images for: Genome-wide transcriptome analysis of gametophyte development in Physcomitrella patens
Source: BMC Plant Biol. 2011 Dec 15;11:177. doi: 10.1186/1471-2229-11-177 (PMC3264550; doi:10.1186/1471-2229-11-177)

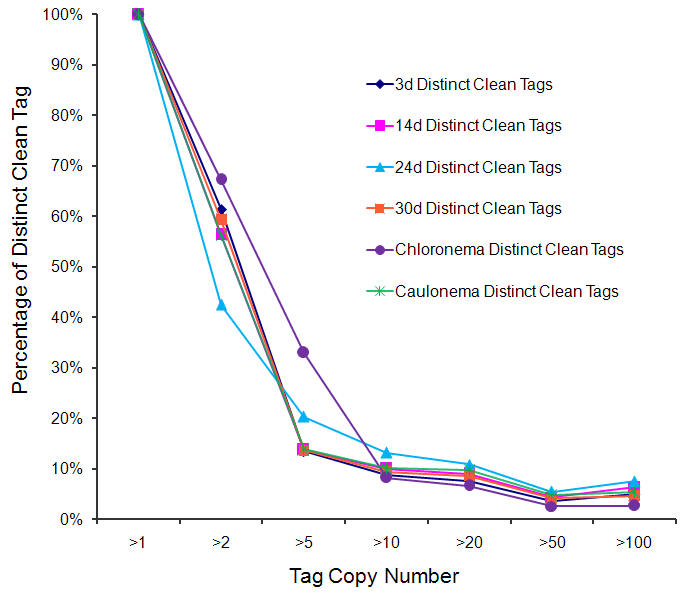

Supplement: Additional file 2 — Distribution of distinct clean tags in each sample. [file 1471-2229-11-177-S2.TIFF]

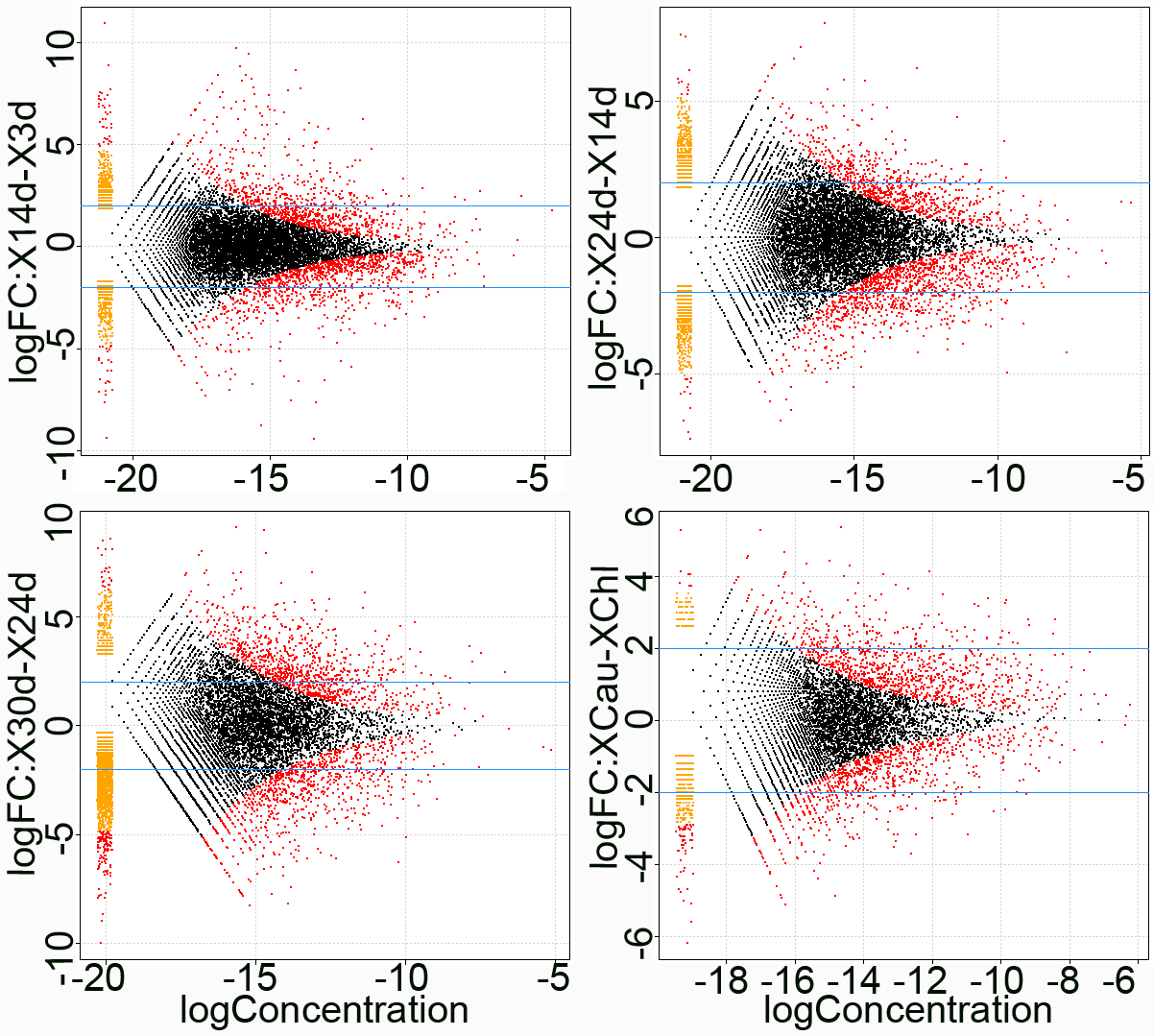

Supplement: Additional file 3 — Smear plots from the edgeR-based analysis of gene expression. Genes are plotted based on their log-fold change of transcript abundance between two compared samples on the y-axis and log concentration on the x-axis for raw tag libraries separately. Differentially expressed genes are shown in red. [file 1471-2229-11-177-S3.TIFF]
