# Supplementary material for: Genome-wide transcriptome analysis of gametophyte development in Physcomitrella patens
Source: BMC Plant Biol. 2011 Dec 15;11:177. doi: 10.1186/1471-2229-11-177 (PMC3264550; doi:10.1186/1471-2229-11-177)
Supplement: Additional file 6 — The primer sequences of the randomly selected and internal genes used for real-time PCR analysis. [file 1471-2229-11-177-S6.DOC]

Additional file 5. The primer sequences of the random selected genes for Real-Time PCR.

*^a^ internal control for 3-day to 30-day’s samples;

| gene ID | sense primer | reverse primer |
| --- | --- | --- |
| Pp1s156_120V6.1 | TGAAGCCTGTGACTGGTG | GCTGCGAACTCAAATCC |
| Pp1s130_191V6.3 | TGTGCCCTCACCGTTTAT | CCACTTCCTTCTCCGTCTT |
| Pp1s154_131V6.1 | GCGGGTTCGCACATTAC | AGGGCAGTTACAGGGTTGA |
| Pp1s170_67V6.1 | GGGAGGGAGTGATGGAGT | TGCTGTGAATGCGTATGG |
| Pp1s20_229V6.1 | GCGTCTGATGAACCTGGGATG | AGTGGGCGGGCTTGTATGG |
| *^a^ Pp1s40_169V6.1 | GCCGTTGAAGGGTCTA | AGGACTCCGACTTAGGTTA |
| *^b^ Pp1s17_377V6.1 | AACGGCTGTGGCTGTGA | TGCCTGATGGCGTAAAT |

*^b^ internal control for chloronema and caulonema samples.
