# Supplementary material for: Genome-wide transcriptome analysis of gametophyte development in Physcomitrella patens
Source: BMC Plant Biol. 2011 Dec 15;11:177. doi: 10.1186/1471-2229-11-177 (PMC3264550; doi:10.1186/1471-2229-11-177)
Supplement: Additional file 7 — Expression level of internal genes in each sample in the real-time PCR analysis. [file 1471-2229-11-177-S7.DOC]

Additional file 7. Expression level of real-time PCR internal genes in each sample.

| gene ID | 3d  (raw tag) | 14d  (raw tag) | 24d  (raw tag) | 30d  (raw tag) | Chloronema  (raw tag) | Caulonema  (raw tag) | 3d  (tags/TPM) | 14d  (tags/TPM) | 24d  (tags/TPM) | 30d  (tags/TPM) | Chloronema  (tags/TPM) | Caulonema  (tags/TPM) |
| --- | --- | --- | --- | --- | --- | --- | --- | --- | --- | --- | --- | --- |
| *^a^ Pp1s40_169V6.1 | 3959 | 4233 | 3825 | 1810 |  |  | 528.84 | 514.77 | 475.84 | 511.71 |  |  |
| *^b^ Pp1s17_377V6.1 |  |  |  |  | 2006 | 2084 |  |  |  |  | 610.74 | 612.5 |

*a internal control for 3-day to 30-day’s samples;

*b internal control for chloronema and caulonema samples.
